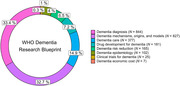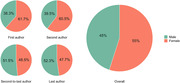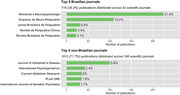# The landscape of dementia research in Brazil (2010‐2021): A Scopus‐based bibliometric study

**DOI:** 10.1002/alz.091819

**Published:** 2025-01-09

**Authors:** Ari Alex Ramos, Laiss Bertola, Fabiana A F da Mata, Andrew Christopher Miguel, Haliton Alves de Oliveira Júnior, Cleusa P Ferri

**Affiliations:** ^1^ Hospital Alemão Oswaldo Cruz, São Paulo, São Paulo Brazil; ^2^ Universidade Federal de São Paulo (UNIFESP), São Paulo, São Paulo Brazil; ^3^ Universidade Federal de São Paulo, São Paulo, São Paulo Brazil; ^4^ Universidade Federal de São Paulo (UNIFESP), São Paulo, São Paulo/SP Brazil

## Abstract

**Background:**

In the last two decades, several studies have sought to investigate the trajectory of scholarly publications on dementia. Yet, there has been limited attention to contributions from Latin America. This bibliometric review aimed to partially address this gap by providing a comprehensive overview of the literature output on dementia in Brazil. Moreover, we were particularly interested in categorizing each included publication according to the research areas in dementia recently defined by the World Health Organization.

**Method:**

We conducted a Scopus‐based literature search for publications indexed between 2010 and 2021 and developed syntaxes into R software for data analysis.

**Result:**

Among the 5,534 documents found in Scopus, 2,528 met the inclusion criteria. The annual growth rate of publications on dementia (9.9%, *SD* = 15.5) closely paralleled that of general health‐related literature (6.7%, *SD* = 4.9), with no statistically significant differences between the two rates. Over 65% of the publications were categorized into the areas of dementia diagnosis (33.4%) and disease mechanisms, origins, and models (32.7%), followed by dementia care (14.9%), drug development (7.2%), risk reduction (6.5%), and epidemiology (4%). There were notably low percentages of publications related to clinical trials (1%) and economic cost (0.3%). Based on the first affiliation of Brazil‐affiliated authors with leading authorship roles (first, second, second‐to‐last, and/or last authors), approximately 90% of the analyzed affiliations stemmed from the Southeast (68.4%) and South (20.9%). However, the State of São Paulo accounted for 41.1%, contributing to 60.1% of the Southeast. Interestingly, 55% of authors holding leading authorship roles were women. Furthermore, first and second authorships were predominantly held by female researchers, whereas male researchers occupied most of the second‐to‐last and last authorships. Overall, 71.7% of the included reports were published in 346 foreign journals and 28.3% in 43 Brazilian journals. The *National Council for Scientific and Technological Development* (CNPq, 19.5%) and the *Coordination for the Improvement of Higher Education Personnel* (CAPES, 11.8%) were the leading research funding agencies acknowledged in the analyzed publications.

**Conclusion:**

There is a pressing need for more studies across all Brazilian regions and all areas of dementia research, especially in epidemiology and economic cost.